# Usefulness of Integrase resistance testing in proviral HIV-1 DNA in patients with Raltegravir prior failure

**DOI:** 10.1186/s12879-016-1545-8

**Published:** 2016-05-13

**Authors:** Jose Ángel Fernández-Caballero, Natalia Chueca, Marta Álvarez, María Dolores Mérida, Josefa López, José Antonio Sánchez, David Vinuesa, María Ángeles Martínez, José Hernández, Federico García

**Affiliations:** Complejo Hospitalario Universitario Granada. Servicio de Microbiología, Hospital Universitario San Cecilio. Instituto de Investigación Ibs. Granada, Av. Del Conocimiento s/n, 18016 Granada (Andalucía), Spain; Domicilio: C/Divan del Tamarit, 4, CP: 18198 Huetor Vega (Granada), Spain

**Keywords:** HIV, Integrase, Proviral DNA, Raltegravir, Dolutegravir

## Abstract

**Background:**

In our study, we have hypothesized that proviral DNA may show the history of mutations that emerged at previous failures to a Raltegravir containing regimen, in patients who are currently undetectable and candidates to simplification to a Dolutegravir containing regimen, in order to decide on once a day or twice a day dosing.

**Methods:**

We have performed a pilot, observational, retrospective, non interventional study, including 7 patients infected by HIV-1, all with a history of previous failure to a RAL containing regimen, that were successfully salvaged and had reached viral suppression. A genotypic viral Integrase region study was available for each patient at the moment of RAL failure. After an average (IQR) time of 48 months (29–53) Integrase resistance mutations in proviral DNA were studied.

**Results:**

All the patients were infected by HIV-1 B subtypes, with a mean age of 55 (range 43 to 56), originating from Spain, and 4 were women. Median viral load (log) and CD4 count at the moment of the study on proviral DNA was of 1.3 log cp/ml (range 0–1.47) and 765.5 cells/μL (range; 436.75–1023.75). The median time (IQR) between previous failure to RAL and the study on proviral DNA was 48 (29–53) months. At Raltegravir failure, N155H was detected in four patients, and other secondary mutations were detected in five patients (71.4 %). In proviral DNA, N155H was detected by population sequencing in three patients (42.8 %), and UDS demonstrated a 9.77 % relative abundance of N155H in the remaining patient. Sanger sequencing correctly identified all the secondary mutations.

**Conclusion:**

This is a pilot study that demonstrates the possibility of properly identifying N155H and some secondary mutations 29–53 months after failure.

## Background

For the last 6 years, Raltegravir (RAL), the first strand Integrase transfer inhibitor (INIs) approved, has played a relevant role on the treatment of HIV infection, both for naive and pretreated patients [1].

Nowadays, Elvitegravir (EVG) and Dolutegravir (DTG), two new INIs have become available. EVG is a first generation drug and shares its resistance profile with RAL, whereas DTG is a second generation INI with a high antiviral effect, and excellent efficacy and safety profile. Although DTG has a high genetic barrier to resistance, its activity may be limited by certain combination of resistance mutations that may accumulate during failure to RAL [2]. The ocurrence of these mutations is not frequent [3] and the use of DTG to salvage patients who have developed resistance to RAL has been well studied and documented [4, 5]. DTG has a long plasmatic half-life, so it can be dosed once a day (QD) on patients without pre-existent resistance to INIs; if the patient to be treated is infected by viruses carrying any resistance mutation to INIs, then DTG has to be dosed twice a day [6, 7] (BID). If the resistance profile at failure was not documented, patients with a prior failure to RAL need to be treated BID, as this is the safest approach.

In our study, we have hypothesized that proviral DNA may show the history of mutations that emerged at failure to RAL, and we attempted to provide the proof of concept that testing proviral DNA may be used as a sentinel for RAL mutations, in patients who are currently undetectable and candidates to simplification to a DTG containing regimen.

## Methods

We have performed a pilot, observational, retrospective, non interventional study, including 7 patients infected by HIV-1, all with a history of previous failure to a RAL containing regimen, that were successfully salvaged and had reached viral suppression. A genotypic viral Integrase region study was available for each patient at the moment of RAL failure. After an average (IQR) time of 48 months (29–53), Integrase resistance mutations in proviral DNA were studied.

Peripheral Blood Mononuclear Cells (PBMC) were separated by Ficoll gradient centrifugation, washed, counted and pelleted to 5x10^6^ leukocytes aliquots, that were used for DNA extraction with QIAamp DSP DNA Blood Mini Kit (QIAGEN). DNA was quantified by spectrophotometry (NanoDrop, ThermoScientific).

Integrase was amplified using a nested-PCR. For the first round of amplification, we used outer primers IN1F (5′-GGAAAAGGTCTACCTGTCATGGGT-3′) and IN1R (5′- GGAGAAAGAGACTGGCATTTGG-3′) and the following PCR profile: 94 °C/2′; (94 °C/15′′; 60 °C/30′′; 72 °C/45′′) x35 cycles; 72 °C/7′; 4 °C. This profile was used for the nested PCR, using inner primers INR2F (5′- TGGAGGAAATGAACAAGTAGATAAATT-3′) and (INT2R: 5′-GGGTCTGCATACAGGAGAAA-3′).

For Sanger sequencing, we used a bidirectional sequencing protocol and the Thermo Sequenase Dye Primer Manual Cycle Sequencing Kit (Affymetrix USB), which was optimized for the TruGene/OpenGene DNA sequencing system (SIEMENS); sequencing primers were P1F:5′- Cy5.5-GTAGCCAGCTGTGATAAATGTC-3′) and P2R (5′- Cy5-CTGCCATTTGTACTGCTGTCT-3′), allowing to sequence 414 nucleotides in the Integrase region, covering positions 40 to 178. The amplification profile was: 94 °C/5′; (94 °C/20′′; 62 °C/20′′; 72 °C/2′) x30 cicles; 70 °C/5′; 4 °C. The obtained sequences were aligned and combined using OpenGene Geneobjects™ software, and fasta sequences were interpreted Stanford HIV Database Versión 7.0 [8].

Ultra Deep Sequencing (UDS) was done using an HIV-1 UDS prototype (Roche diagnostics) and the 454 GS Junior (Roche 454 Life Sciences Branford, CT). As template for the UDS PCR, we used the same amplification products as for standard Sanger sequencing following from this point the manufacturer’s recommendations. Once UDS was performed, sequences were exported by AVA (GS Amplicon Variant Analyzer, Roche) software and interpreted by DeepChek®- VIH (ABL, SA) software, using for interpretation the same Stanford version as for Sanger sequencing.

This was a retrospective, non-interventional study, and patient information was anonymized and de-identified prior to analysis.

## Results

All the patients were infected by HIV-1 B subtypes, with a mean age of 55 (range 43 to 56), originating from Spain, and 4 were women. Median viral load (log) and CD4 count at the moment of the study on proviral DNA was 1.3 log cp/ml (range 0–1.47) and 765.5 cells/μL (range; 436.75-1023.75). The median time (IQR) between previous failure to RAL and the study on proviral DNA was 48 (29–53) months. Detailed information on baseline characteristics is shown in Table 1.

**Table 1:** Socio-demographic and clinical characteristics of patients

Patient	Date of failure	Age	CD4	VL (log)	Time (monghts) among samples
1	15/10/2009	56	NR	ND	21
2	20/10/2009	56	484	1,47	53
3	01/10/2009	59	670	1,30	52
4	17/02/2011	39	1365	1,47	36
5	02/03/2010	51	910	1,30	48
6	26/10/2012	43	295	2,74	29
7	13/8/2009	55	861	ND	56

Viral load (VL) is in the actual moment. (*NR* Not recorded, *ND* Not detectable)

Table 2 shows the correlation on resistance mutations (Sanger sequencing) detected at RAL failure in plasma and on proviral DNA, after a median period of 48 months of being undetectable. At failure, N155H was detected in four patients, and other secondary mutations were detected in five patients (71.4 %). In proviral DNA, N155H was detected by population sequencing in three patients (42.8 %), and UDS demonstrated a 9.77 % relative abundance of N155H in the remaining patient. Sanger sequencing correctly identified all the secondary mutations. We observed that proviral DNA and plasma RNA drug resistance mutations and polymorphisms were highly concordant.

**Table 2:** Primary and secondary resistance mutations in the Integrase by Sanger population sequencing

Patient	Major resistance mutation	Accesory mutation	Polymorphism mutation
1F	N155H	__	C56S, E85EG, L101I, S119P, T122I, H171Q, K173EK
1A	N155H	__	C56S, L101I, S119P, T122I, H171Q
2F	N155H	__	M50I, L68R, V71I, L101I, S119P, H171Q
2A	N155H	__	M50I, V71I, P90PS, L101I, S119P
3F	__	L74I	E96D, K111T, K160KT
3A	__	L74I	E96D, K111T, G123RS
4F	__	G163GR	L101I, I113V, G134E, V150AV
4A	__	G163GR	M50IM, L101I, I113V, V150A
5F	__	L74IM	M50V, V72I, K103R, K111T, A124T
5A	__	L74I	M50V, V72I, K103R, K111T
6F	N155H	T97A	D55Y, V72I, K111T, I113V, S119R, G123S, A124N, T125A
6A	__	__	V72I, K111T, I113V, S119R, G123S, A124N, T125A
6 UDS	N155H (9.77 %)	T97A (12.42 %)	V72I(37.44 %), Y99C(4.65 %), T122I(12.56 %), K156N(14.35 %), E157A(15.35 %), K111T(39.53 %), I113V(29.3 %), S119R(37.44 %), G123S(97.21 %), A124N(43.26 %), T125A(45.58 %)
7F	N155H	V151I	I113V, S119P, T122I, A124N, C130Y
7A	N155H	V151I	G52P, S119PR, T122I, I161X

Patients are indicated with the numbers 1 to 7; F relates to the time point of therapeutic failure (plasma RNA), A to the proviral DNA studies after virological suppression, and UDS to massive sequencing data

## Discussion

Dolutegravir has shown excellent efficacy and safety in individuals infected by HIV both in naïve [9], and patients with prior exposure to RAL [10]. Only the accumulation of Q148H/R/K, together with other secondary mutations broadens DTG activity [11]. The VIKING study evaluated Dolutegravir dosing, demonstrating a higher efficacy, tolerability and safety when dosing DTG 50 mg [10] twice a day (BID) for patients with resistance mutations in the Integrase. While BID is the safest approach, DTG is only recommended 50 mg once a day (QD) for patients with no resistance against Integrase inhibitors. For some patients who have not been tested for Integrase resistance at failure, and have been effectively suppressed with a new antiretroviral regimen, BID remains the safest dosing strategy, but QD could possibly play a role, reducing the cost of the new regimen.

Proviral DNA may be a useful tool to investigate the presence of resistance mutations [12–14], especially in patients who as a consequence of antiretroviral therapy are virologically suppressed.

In our study, using Sanger sequencing of the Integrase region of proviral DNA, we could correctly identify failing selected mutations in 6/7 patients. Although for the remaining patient we could not demonstrate the failing mutation with Sanger sequencing, using a more sensitive test [15], resulted in the correct identification of the failing mutations [N155H (9.7 %) and T97A (12.42 %)] suggesting that, given the superiority of massive parallel sequencing, this should be the tool recommended for testing proviral DNA in virologically suppressed patients, although at present it is an expensive tool that may not be feasible in some laboratories.

Although the sampling time in patients 2, 3 & 7 exceeded the half-life of the HIV-1 reservoir, this did not interfere in the correlation between the failing sample and proviral DNA testing. Despite some studies have demonstrated that the latent viral reservoirs half-life is from four to six months in patients who start therapy in the acute infection stage, there are other studies in chronically infected patients who have shown a half-time of 44 months [16, 17].

Our study has certain limitations. First, only subtype B patients have been included, so the methodology needs to be validated for other subtypes. Secondly, only the N155H pathway was confirmed in some patients and it is possible that resistance pathways other than N155H, that could have emerged before N155H was established, may have been archived in the proviral DNA of the patients compromising DTG activity.

## Conclusions

In summary, despite the limitations of our study, which is just a pilot study that should be confirmed in further studies, we have shown the proof of concept that for patients who failed a Raltegravir containing regimen in the past, who are currently virologically suppressed, and lack the resistance information at failure, studying Integrase resistance in the proviral DNA accurately reflects the possibility of properly identifying N155H and some secondary mutations 29–53 months after failure.

## Ethics approval

The Ethics Committee of the San Cecilio Hospital approved the study, and no consent information was required as patient information was anonymised and de-identified prior to analyses.

## Consent for publication

Not applicable.

## Availability of data and materials

All data supporting our findings is contained within the manuscript.

## Abbreviations

AVA: amplicon variant analyzer
BID: twice a day
DNA: deoxyribonucleic acid
DTG: Dolutegravir
EVG: Elvitegravir
HIV: human immunodeficiency virus
INIs: strand integrase inhibitor
IQR: interquartile range
PBMC: peripheral blood mononuclear cells
PCR: polymerase chain reaction
QD: once a day
RAL: Raltegravir
UDS: ultra deep sequencing
